# Multidisciplinary endeavors make future medicine smart

**DOI:** 10.1002/SMMD.20220031

**Published:** 2022-12-23

**Authors:** Luoran Shang, Yihai Cao, David A. Weitz

**Affiliations:** ^1^ Institutes of Biomedical Sciences Fudan University Shanghai China; ^2^ Department of Microbiology, Tumor and Cell Biology Karolinska Institutet Solna Sweden; ^3^ John A. Paulson School of Engineering and Applied Sciences Harvard University Cambridge Massachusetts USA; ^4^ Department of Physics Harvard University Cambridge Massachusetts USA

Humans suffer from thousands of diseases, and many of them lack effective methods for diagnosis and treatment. To address these unmet medical needs, basic research in multiple areas can provide important clinical contributions. The breathtaking discoveries in fundamental life sciences not only enhance our understanding of the human body but also lead to the creation of new diagnostics and pharmaceuticals. Innovations in medically relevant technologies, drawing on a wide range of disciplines, bring hope to previously intractable illnesses. All these lead to the dramatic transformation of medicine toward “Smart Medicine.” We foresee the future of medicine to be nourished with cutting‐edge ideas from many different fields. We also anticipate specially designed methods that help translate these ideas and make them fit for specific healthcare purposes.

The launching of *Smart Medicine* is rooted in the idea that there is an urgent need for a journal that embraces all medically relevant research. *Smart Medicine* is copublished by Wiley and the Wenzhou Institute of the University of Chinese Academy of Sciences (WIUCAS, China). It aims to foster communications among and joint efforts by multiple science and engineering communities, thus sparking new ideas in the medical arena. *Smart Medicine* is overseen by a world‐class editorial group with members from different research backgrounds. We proudly introduce our Editors‐in‐Chief professors, Prof. Yihai Cao (*Karolinska Institute, Sweden*) and Prof. David A. Weitz (*Harvard University, USA*), our Executive Editors, Prof. Wenguo Cui (*Shanghai Jiao Tong University, China*), Prof. Hongbo Zhang (*Åbo Akademi University, Finland*), and Prof. Yuanjin Zhao (*Southeast University, China*), our Associate Editors, Prof. Fangfu Ye (*WIUCAS, China*), Prof. Cecilia Sahlgren (*Eindhoven University of Technology, Netherlands*), Prof. Kenneth L. Pitter (*Ohio State University, USA*), and Prof. Sergey Filippov (*University of Reading, UK*), and 28 Editorial Board members coming from 16 countries or regions. We would also like to thank the full support from the professional publishing team of Wiley, Dr. José Oliveira, Dr. Guangchen Xu, Dr. Jing Zhu, Dr. Xiaoyu Zhang, and Ms. Kelsey Ma.

In this inaugural issue of *Smart Medicine*, we have collected 4 research articles, 1 minireview, and 7 review papers focusing on different aspects of basic, clinical, and translational medicine. The original studies include that led by Prof. Haozhen Ren (*Nanjing Drum Tower Hospital, China*) on liver lobule‐mimicking scaffolds for tissue engineering, Prof. Fangfu Ye on structural color films for biosensing, Prof. Qingming Ma (*Qingdao University, China*) on bacterial infection microenvironment‐responsive microspheres for anti‐infective therapy, and Prof. Hongbo Zhang on reregulated mitochondrial dysfunction reversing cisplatin resistance microenvironment in colorectal cancer. The single minireview contributed by Prof. Feili Lai's group (*KU Leuven, Belgium*) focuses on personalized healthcare. Additionally, we have invited Prof. Yuanjin Zhao, Prof. Veijo Hukkanen (*University of Turku, Finland*), Prof. Wenguo Cui, Prof. Renjie Chai (*Southeast University, China*), Dr. Lei Tian (*McMaster University, Canada*), Prof. David A. Posner (*University of Cambridge, UK*), and Prof. Luoran Shang (*Fudan University, China*) to contribute authoritative reviews on several thriving medically relevant technologies and engineering topics, including microfluidics, drug development, reprogramming stem cells, functional hydrogels, living materials, combination therapies, and biomimetics.

We hope this inaugural issue will help readers appreciate the multidisciplinary principles, broad scope, and high‐quality standards of *Smart Medicine*. We also hope that our readers will consider *Smart Medicine* as among the most preferred journals in which to share exciting research results and forward‐looking viewpoints. The future of medicine has come; let us explore a huge world of new possibilities with *Smart Medicine*.

## CONFLICT OF INTEREST

The authors declare that they have no conflict of interest. Luoran Shang, Yihai Cao, and David A. Weitz are members of the *Smart Medicine* editorial board.

